# A Case Series of Severe Dengue with Neurological Presentation in Children from a Colombian Hyperendemic Area

**DOI:** 10.1155/2021/6643738

**Published:** 2021-06-01

**Authors:** Jaime E. Castellanos, Paula Esteban, Juanita Panqueba-Salgado, Daniela Benavides-del-Castillo, Valentina Pastrana, Gladys Acosta, Doris Salgado, Carlos F. Narvaez, Sigrid Camacho-Ortega, Eliana Calvo, Myriam L. Velandia-Romero

**Affiliations:** ^1^Grupo de Virología, Universidad El Bosque, Bogotá, Colombia; ^2^Facultad de Medicina, Universidad El Bosque, Bogotá, Colombia; ^3^Grupo de Parasitología y Medicina Tropical, Universidad Surcolombiana, Neiva, Colombia

## Abstract

Dengue transmission is sustained in Colombia with increasing prevalence mainly in children. This work aimed to describe a case series of children diagnosed with dengue presenting neurological disease in Huila Province of Colombia. Eleven pediatric febrile patients confirmed for dengue disease and presenting neurological signs were studied in the University Hospital of Neiva, Huila Province. Clinical and laboratory findings, CSF cytochemical analysis, neurology images, and serology and molecular studies were performed. Viral RNA was detected in all patients' sera by RT-PCR. Nine out of 11 were primary infections. Tonic-clonic seizures (73%), consciousness alterations (27%), irritability (27%), and ataxia (18%) were the most frequent neurological signs. None of the patients had plasma leakage, hypovolemic shock, or liver disease, confirming the encephalitis diagnosis. Diagnostic images did not show abnormal findings, but neither bacterial nor fungal infections were detected in CSF analysis. All patients survived without sequelae except for one patient that presented ataxia for months. In conclusion, we described a group of children with neurological signs during severe dengue disease as the main finding, indicating the importance to including dengue as a differential diagnosis in neurological patients from endemic areas.

## 1. Introduction

The world estimate of dengue infections is around 390 million people among symptomatic and asymptomatic cases in endemic countries such as those of Latin America and the Asian, Southeast, and Pacific Islands [1]. Nearly 65% of Colombian municipalities reported constant *Aedes* infestation [2] and dengue incidence increase year after year mainly associated with abnormal environmental sanitary conditions, broad circulation of the four serotypes, and high mosquito infestation [3, 4]. In America, most of the cases occur in individuals under 15 years of age [5].

The largest dengue occurred in Latin America was reported in 2019 with more than three million cases [6] reaching an incidence rate of 80 cases per 100,000 inhabitants.

The current disease classification propose is to identify dengue following clinical signs and symptoms to establish the most accurate diagnosis and recognize early cases that could become severe or fatal [7]. Accordingly, dengue warning signs reflecting endothelial dysfunction that should be revised are abdominal tenderness, vomit, edema, mucosal bleeding, hepatomegaly, irritability, or hypotension. In those presumptive dengue cases occurring with plasma leakage signs (pleural effusion and pericarditis) or respiratory distress, severe bleeding, or affectation of organs such as the liver, brain, or kidney, a diagnostic of severe dengue should be corroborated [8].

Even although neurological manifestations were reported since 1976 in Thailand as “dengue-associated acute encephalopathy” [9], from these years until today, the incidence of neurologic manifestations during dengue disease has increased considerably [10]. Similar cases have been reported from the Asian, Southeast, and Pacific Islands of America [11, 12]. Today, some hypotheses proposes that DENV could also be neurotropic as other flaviviruses supported by the frequent finding of specific IgM or viral RNA in the cerebrospinal fluid (CSF) [13, 14] that could confirm CNS infection and direct brain injury. This finding allows the differentiation of those circulating viruses and antibodies that could enter to the CNS due to impairment of the blood-brain barrier (BBB).

Many kinds of pathologies have been reported, from cerebral edema and hemorrhage to perivascular lymphocyte infiltrates and demyelination foci. These anomalies could be explained by i) BBB alterations induced by TNF-alpha and IL-6 activity involved in endothelial junction disruption favoring virus entry to the CNS, ii) DENV infection to endothelial cells leading to virion pass through to brain parenchyma [15, 16], and iii) endothelial cell injury by glycocalyx remodeling induced by NS1. Nervous system DENV replication has been observed in the olivary nucleus and cerebellum as well as tissue macrophages DENV positive, indicating some neurotropism [17].

It has been reported that 47% of adult meningitis and encephalitis cases in a Brazilian endemic area were explained by acute DENV infection occurring during the first week of fever onset [18]. During that outbreak, the disorder did appear as encephalitis with behavioral disorders, seizures, and paresis with no evidence of liver failure and confirming serum or CSF IgM serology or altered neuroimages showing brain changes. Other cases with unspecific neurological signs could appear as a consequence of metabolic alterations or prolonged shock, brain hypoxia, cerebral edema, or liver or kidney failure that explain the headache, retro-orbital pain, insomnia, restlessness, and mood changes appearing in patients [19]. The Colombian study reported by Mendez and Gonzalez [20] described 46 out of 168 severe dengue child patients presenting encephalopathy and 3 with encephalitis diagnostic. Dengue encephalitis is considered when febrile patients in endemic areas, having one of these signs or symptoms, headache, seizures, consciousness alterations, and behavioral changes with no hepatic failure or bleeding and positive for DENV serology or molecular test in serum or CSF with altered brain images in tomography or resonance. The purpose of this article is to present and describe the clinical, laboratory, and diagnostic characteristics of eleven children diagnosed as dengue cases, which presented neurological manifestations, cases occurring during regular endemic circulation in the Colombian province of Huila in 2011. DENV spreading in Latin-American countries and inside of them has been explained by air traffic between cities, urban population size, and human development index, which predict positively the outbreaks in those endemic cities like Neiva studied here (Allicock).

## 2. Cases Presentation

The University Hospital Hernando Moncaleano is the largest health setting in Neiva, the capital city of Huila Province, located at 42 meters above sea level with a mean temperature of 28°C where it has been reported frequent dengue outbreaks. The Hospital Institutional Review Board approved the study and parents or guardians signed the consent form during the stay. We collected the clinical and laboratory information of those febrile patients with one of dengue symptoms such as rash, headache, retro-orbital pain, myalgia, and arthralgia associated with any neurological signs such as seizures, abnormal movements, acute flaccid palsy, lethargy, meningeal signs, or consciousness alteration with no shock evidence, which were diagnosed as severe dengue following the recommendation of the WHO. Clinical records from the pediatric emergency department were revised from March to October 2011 and those patients with neurological signs and another microorganism associated were excluded. One specific designed clinical report form was filled for each case, and complete information of laboratory tests (blood cell counts, coagulation tests, liver, and kidney function markers), diagnostic images (tomography and chest Rx), and CSF chemical and microbiological analysis was collected; however, other viral pathogens were not evaluated. Sera from the enrolled cases were processed for serum IgM and capture IgG detection by ELISA and for viral RNA detection by RT-PCR on sera following the protocol described previously [21].

During the study period, 34 patients with fever and neurological manifestations were examined, but three were excluded (one by mental retardation and two with psychiatric disorders); the remaining 31 patients were investigated for dengue by serology or sera RT-PCR, and eleven (35.5%) tested positive for any of them (two girls and nine boys). All of them, finally, were discharged with severe dengue diagnostic or viral encephalitis, despite all of them having positive dengue IgM or IgG test in serum. All but one patient had laboratory analysis, although this one had serology and RT-PCR results. Most of the patients (73%) presented seizure disorder or convulsions. In Table 1, the complete data of patients are shown. Although all patients were IgM positive, only two were IgG positive (secondary infection). All sera were positive for RT-PCR for DENV, but only three of them allowed serotyping (DENV-1).

Clinically, eight patients presented tonic-clonic seizures although only 2/11 reported headache and three developed consciousness alteration or irritability, two had ataxia symptoms, and one developed unilateral paresis. Other signs found were vomiting (5), hepatomegaly (5), cough (5), abdominal pain (3), asthenia (3), and epistaxis (2). None of the patients presented plasma leakage signs such as edema or pleural effusion, nor altered transaminase values. There was brain tomography in eight patients and one magnetic resonance, all showing no alterations. Additionally, chest Rx from three patients was normal. Despite the severity, there was no fatal case, and all of them, but one, recovered without sequelae.

The hematimetric analysis indicated anemia and low hematocrit in all patients (median 33.5%, IQR 31.8-34.6%) since the child normal value is 36–42%. A mild leukocytosis was observed at day one and a decrease until day four when leukopenia was evident. Six patients developed thrombocytopenia with the day 6 value being the lowest (median 99.200 platelets/mm^3^). The coagulation prothrombin test only showed a mean of 15.4 s; it means a prolongation value at the first hospitalization day. However, the partial thromboplastin time (PTT) was prolonged during six hospitalization days with values between 32.3 and 35.1 s (Table 2). Transaminase levels used to evaluate the hepatic function and AST showed a slight increase during the last three hospitalization days (fifth to the seventh day), but the values were close to those reference levels while ALT did not show changed levels. There were no changes in renal function markers in any of the patients.

## 3. Discussion

Colombia has seen an increase in the dengue cases number in the last decade with higher severity and mortality [4, 5], forcing medical personnel and health authorities to strengthen capacity to recognize and differentiate classical dengue signs and symptoms from the atypical and severe presentation. After the redefinition of diagnostic and severity criteria by the WHO [22], severe dengue is defined when patients present organ damage, associated signs (liver, brain, and kidney), or hypovolemic shock or massive bleeding. This system facilitates the early recognition of the most severe cases by reducing the mortality numbers.

This study presented eleven severe dengue cases in children between eight months to 10 years of age, living in the Colombian province Huila, a hyperendemic area that reported two-fifths of severe dengue cases registered from 2000 to 2011 [5]. All patients presented brain compromise; nine were diagnosed as not only encephalitis (81.8%) following the criteria described previously [23], such as fever, sensory disturbances, seizures, or focal neurological signs, but also positive tests (IgM or NS1) or RT-PCR in serum or CSF and negative for others encephalitis causes. This presentation was the most common compromise in this series, characterized by seizures in six patients (tonic-clonic convulsions) as has been previously reported in children [24]. There was one severe sensory impairment case, and another presented with severe headache, which also is frequently described in dengue encephalitis [25]. Less than half of the described patients develop classical dengue signs such as vomiting, hepatomegaly, and exanthem, indicating the neurological cases do not seem to have dengue disease and that nervous signs could appear alone leading to registration of severe cases and possibly in the delay of the establishment of adequate treatment [23]. Two patients were diagnosed as cerebellitis, characterized by nystagmus, dysarthria, or ataxia, which also has been reported previously [26]. Fortunately, there were no fatal cases in this patient group; on the contrary, outcomes and recovery were satisfactory.

It is recommended that, in DENV circulation areas, those patients with fever and neurologic signs should be investigated for dengue disease, ensuring laboratory confirmation using both serology and molecular tests that, together, could offer high sensitivity [26]. Here, we used both IgM and IgG serology in addition to RT-PCR and found all serum samples positive, even serotyping DENV-1 in three patients, which is not the most reported associated with neuroinfection [27]. The finding of nine sera negative for IgG is very odd because most of the severe dengue cases are during secondary infections with a different serotype, where the antibodies are not capable of the second virus neutralization but induce an enhanced infection and an aberrant immune response. Therefore, severe dengue with a primary compromise of an organ in primoinfection could suggest a neurotropic strain or a host characteristic that determined the neuroinfection in this child group.

All CSF samples were negative for bacteria or fungi, but five out of nine had lymphocyte pleocytosis, an uncommon sign in dengue encephalitis; since most of the cases do not develop CSF anomalies [24], even three-quarters of dengue encephalitis or meningitis patients showed normal values in CSF analysis [27]. Despite previous studies reported that brain tissue damage is detectable by tomography images, the children described in this work did not present anomalies. Although it is preferable to use magnetic resonance for brain evaluation because of the accuracy of this technique, regularly imaging abnormalities during dengue encephalitis are not specific [28] and are still poorly defined [29, 30], although ischemia or hemorrhage must be sought [31]. On the other hand, we found normal leukocyte counts instead of the classical leukopenia of dengue with warning signs or severe dengue, which pose a difficulty to early diagnosis, although there was thrombocytopenia only at day six of hospitalization with no changes in ALT and AST transaminases as reported previously. Recently, 11/36 patients from a Brazilian endemic area were diagnosed with neurologic manifestations and confirmed for arbovirus infection using CSF or sera [32] (5 DENV and 6 CHIKV), posing an additional warning about involvement of these viruses in severe disease presentation in Latin-American countries where there is cocirculation.

## 4. Conclusions

This child group were diagnosed as severe dengue with central nervous system manifestations without shock, typical signs, or hemorrhage. Therefore, we should recommend that, in endemic dengue areas, health professionals should include infections by this virus in those patients with fever and neurological manifestations as part of the primary diagnostic and must be vigilant for neurological signs that could appear after acute dengue disease.

## 5. Limitations

This study was retrospective and is limited by its observational nature. Although we enrolled almost all those children with severe dengue diagnosis in this hospital, detailed clinical and laboratory information was obtained from these reported patients after an in-depth search of data in the medical records.

## Figures and Tables

**Table 1:** Clinical and laboratory findings of evaluated patients.

Code	Age	Clinical findings	Laboratory findings	Images	Presumptive diagnosis	Dengue IgM (serum)	Dengue IgG (serum)	Cerebrospinal fluid analysis	Serum molecular test	Discharge diagnosis
**430**	8 months	Tonic-clonic seizure, fever, rash, and hepatomegaly	Lymphocytic leukocytosis, anemia, thrombocytopenia	Simple and contrasted brain CAT scan with no lesion or midline alteration	Dengue with warning signs and febrile convulsions	Positive	Negative	Clear liquid, with no RBC or leukocytes. Glucose 59 mg/dL and proteins 21.5 mg/dL. Negative test for *H. influezae, S. pneumoniae*, group B *Streptococcus, N. meningitidis, Criptococus neoformans* negative, and *M. tuberculosis* negative	RT-PCR (+)	Severe dengue
**836**	11 months	Four limbs hypotonic, epigastralgia, painful right hypochondrium, neck adenomegaly, and fever	Leukocytosis, anemia, and blood clotting alteration	Simple brain CAT scan with no lesions or hemorrhages	Febrile syndrome	Positive	Negative	Not available	RT-PCR (+)	Severe dengue febrile seizures
**148**	12 months	Fever, tonic-clonic seizures, peri buccal cyanosis, mydriasis, and tachypnea	Anemia	Simple brain CAT scan normal. Normal chest radiography	Convulsive syndrome meningitis	Positive	Negative	Clear liquid, with no RBC or leukocytes. Glucose 63 mg/dL and proteins 22.1 mg/dL. Negative test for *H. influezae, S. pneumoniae*, group B *Streptococcus, N. meningitidis, Criptococus neoformans* negative, and *M. tuberculosis* negative	RT-PCR (+) DENV-1	Severe dengue
**273**	12 months	Fever, generalized tonic convulsion, sialorrhea, and rhonchus	Leukopenia, anemia, and thrombocytopenia	Not done	Convulsive syndrome	Positive	Negative	Cloudy and bloody liquid. Negative for leukocytes. RBC 195.000/mm3, glucose 76 mg/dL, and proteins 72.3 mg/dL. Negative test for *H. influezae, S. pneumoniae*, group B *Streptococcus, N. meningitidis, Criptococus neoformans* negative, and *M. tuberculosis* negative	RT-PCR (+) DENV-1	Severe dengue and dengue encephalitis
**795**	12 months	Tonic-clonic seizures, fever, vomiting, cough, tachypnea, rhonchus, skin and mucosae pallor, and sialorrhea	Leukopenia, anemia, and thrombocytopenia	Not done	Convulsive syndrome acute pharyngitis viral encephalitis	Positive	Negative	Clear liquid, leukocytes 98/mm3, glucose 44 mg/dL, and proteins 64.9 mg/dL. Negative test for *H. influezae, S. pneumoniae*, group B *Streptococcus, N. meningitidis*, *Criptococus neoformans* negative, and *M. tuberculosis* negative	RT-PCR (+)	Severe dengue, bacterial meningitis, viral encephalitis, bronchiolitis, and convulsive syndrome
**941**	5 years	Ataxia, fever, generalized abdominal tenderness, and cough	Normal	Simple brain CAT scan normal	Ataxia and postinfectious cerebellitis	Positive	Negative	Clear liquid, with no RBC, leukocytes 4/mm3, glucose 48 mg/dL, and proteins 25.8 mg/dL. Negative test for *H. influezae, S. pneumoniae*, group B *Streptococcus, N. meningitidis, Criptococus neoformans* negative, and *M. tuberculosis* negative	RT-PCR (+)	Severe dengue, ataxia, and postinfectious cerebellitis
**308**	5 years	Fever, seizures, asthenia, adynamia, rash, petechiae, headache, and vomiting	Leukopenia, thrombocytopenia, leucopenia, and increased ALT value	Chest rx normal and simple brain CAT scan normal	Dengue with warning signs and vasculitis	Positive	Negative	Clear liquid, RBC 220.000/mm3, leukocytes 3000/mm3, glucose 56 mg/dL, and proteins 55.7 mg/dL. Negative test for *H. influezae, S. pneumoniae*, group B *Streptococcus, N. meningitidis, Criptococus neoformans* negative, and *M. tuberculosis* negative	RT-PCR (+)	Severe dengue focal seizures
**925**	7 years	Fever, tonic-clonic seizures, sphincters relaxation, asthenia, adynamia, irritability, vomiting, and hepatomegaly	Thrombocytopenia - PTT prolonged	Simple brain CAT scan normal	Dengue with warning signs. Epilepsy	Positive	Positive	Not done	RT-PCR (+) DENV-1	Severe dengue
**329**	7 years	Fever, vomiting, diarrhea, tachycardia, hypotension, mucosa and skin pallor, hepatomegaly, low blood pressure, respiratory distress (intercostal retractions, nasal flaring, and basal rales), hypoactivity, and stupor	Hematocrit reduction and creatinine value reduction	Brain resonance normal and mastoiditis	Viral encephalitis, gastroenteritis, and acute respiratory infection	Positive	Negative	Clear liquid, with no RBC, leukocytes 720/mm3, glucose 49 mg/dL, and proteins 155 mg/dL. Negative test for *H. influezae, S. pneumoniae*, group B *Streptococcus, N. meningitidis, Criptococus neoformans* negative, and *M. tuberculosis* negative	RT-PCR (+)	Severe dengue viral encephalitis and sepsis
**210**	10 years	Headache, fever, nauseas, ataxia, vomiting, hyporeflexia, epistaxis, asthenia, and irritability	Not available	Simple brain CAT scan with no alteration	Acute ataxia and viral cerebellitis	Positive	Positive	Clear liquid, RBC 20/mm3, leukocytes 90/mm3, glucose 44 mg/dL, and proteins 68 mg/dL. Negative test for *H. influezae, S. pneumoniae*, group B *Streptococcus, N. meningitidis, Criptococus neoformans* negative, and *M. tuberculosis* negative	RT-PCR (+)	Severe dengue. Acute ataxia Bipolar disorder
**797**	10 years	Fever, tonic-clonic seizures, epistaxis, dehydration, and irritability	Leukopenia, thrombocytopenia, PTT prolonged, and AST value increased	Simple brain CAT scan normal, electroencephalogram normal, and chest Rx normal	Convulsive syndrome	Positive	Negative	Clear liquid, with no RBC or leukocytes, glucose 71 mg/dL, and proteins 21.4 mg/dL. Negative test for *H. influezae, S. pneumoniae*, group B *Streptococcus, N. meningitidis, Criptococus neoformans* negative, and *M. tuberculosis* negative	RT-PCR (+)	Severe dengue focal seizures

**Table 2:** Hematologic parameters description according to hospitalization day (mean (IQR) [n]).

	Parameter	Day 1	Day 2	Day 3	Day 4	Day 5	Day 6	Day 7
Blood parameters median (IQR) [*n* = ]	Hemoglobin (mg/dL)	11.5 (11.2–11.9) [7]	10.7 (10.3–11.6) [6]	10.4 (10–10.4) [6]	11.5 (10.9–12.2) [6]	10.8 (9.6–12.2) [5]	11.5 (10.5–13.2) [6]	11.6 (11.4–12.3) [5]
	Hematocrit (%)	33.8 (33.5–36.8) [7]	32.5 (31.1–34.2) [6]	31.8 (31–32.5) [6]	33.9 (32.2–35.5) [6]	32.6 (29.3–35.5) [5]	34.6 (32.6–39.6) [6]	33.5 (33.4–35) [5]
	Leukocytes (x 1000 cell/*μ*L)	9.8 (5.9–13.9) [8]	7.5 (5.1–8.8) [6]	6.8 (4.0–11.1) [7]	3.6 (2.8–4.8) [6]	5.1 (2.7–6.5) [6]	5.7 (1.8–8.4) [6]	8.4 (6.0–7.2) [5]
	Neutrophils (%)	56 (41–71) [8]	45.6 (31–60) [6]	42.5 (30–51) [6]	36.6 (28–43) [6]	38.6 (35–51) [6]	32.6 (18.2–43.4) [6]	35.8 (32–37) [5]
	Lymphocytes (%)	33.9 (19.6–46.8) [8]	47.1 (33–62) [6]	48.9 (87.9–60.3) [6]	52.1 (43–69) [6]	51.8 (36–57) [6]	53.2 (37–67) [6]	40.3 (31–56) [5]
	Platelets (x1000 cell/*μ*L)	184.8 (109–230) [7]	251.8 (130–372) [7]	266.8 (180–380) [6]	199.1(105–207) [6]	169.2 (88–211) [5]	99.2 (80–88) [5]	271.4 (85–457) [5]

Clinical laboratory values mean (SD) [*n* = ]	PT (seg)	^∗^15.4 (2.3) [5]	13.5 [1]	13.8 (0.6) [2]	13.7 (2.7) [3]	14.2 (1.6) [6]	12.7 (1.1) [4]	13.2 (0.5) [3]
	PTT (seg)	^∗^35.1 (8.9) [5]	^∗^37.9 [1]	29.8 (2.4) [2]	^∗^32.9 (5.6) [3]	^∗^32.3 (8.1) [6]	^∗^32.66 (5.7) [3]	^∗^37.1 (1.0) [4]
	AST (UI/mL)	^∗^66.9 (35.9) [3]	^∗^78.9 (14.4) [2]	59.3 [1]	50.4 (21.1) [3]	^∗^82.4 (52.5) [4]	^∗^83.1 (43.4) [4]	^∗^108.5 (78.1) [2]
	ALT (UI/mL)	75.9 (96.6) [3]	90.4 (96.6) [2]	28.2 (12.4) [2]	23.4 (6.1) [3]	35.2 (12.3) [5]	48.3 (20.9) [4]	49.5 (18.1) [3]
	BUN (mg/dL)	4.45 (1.3) [2]	NA	2.75 (0.4) [2]	NA	5.4 (6.1) [2]	3.6 [1]	9.9 (0.1) [2]
	Creatinine (mg/dL)	0.29 [3]	0.38 [1]	0.27 [2]	0.2 [1]	0.36 [3]	0.27 [1]	0.52 [1]

^∗^Significant differences between values in different hospitalization days, NA: not available. PT, prothrombin time. PTT, partial thromboplastin time. AST, aspartate transaminase. ALT, alanine aminotransferase. BUN, blood urea nitrogen.

## Data Availability

The dataset supporting the conclusions of this article is available in the http://www.researchgate.net repository, DOI: 10.13140/RG.2.2.32618.54728 and DOI: 10.13140/RG.2.2.15841.33128.
